# TTN variants in pediatric cardiomyopathy: a retrospective cohort study

**DOI:** 10.3389/fgene.2026.1758524

**Published:** 2026-04-01

**Authors:** Yu Qiu, Lei Liu, Kaiyu Zhou, Yifei Li, Yimin Hua

**Affiliations:** Key Laboratory of Birth Defects and Related Diseases of Women and Children of MOE, Department of Pediatrics, West China Second University Hospital, Sichuan University, Chengdu, Sichuan, China

**Keywords:** dilated cardiomyopathy, pediatric, prognosis, titin, whole-exome sequencing

## Abstract

**Background:**

Titin (TTN) variants have been implicated in various types of cardiomyopathy. Allelic variant heterogeneity results in variable clinical phenotypes, which remains a major barrier for effective disease management. We aim to investigate the relationship between *TTN* variants and their associated cardiomyopathies and clinical outcomes.

**Methods:**

A retrospective observational study was performed to evaluate patients with cardiomyopathy and *TTN* variants confirmed by whole-exome sequencing (WES) from January 2015 to December 2024. Univariable Cox regression analysis was conducted to identify independent risk factors for major adverse cardiovascular events (MACEs), and receiver operating characteristic analysis was used to determine its capability. In addition, the contribution of combined pathogenic variants with the TTN gene was assessed.

**Results:**

A total of 53 patients were identified with TTN variants, with a *median onset age of 42.3 months (IQR 18.5–76.1)*, while 48 of 53 (90.50%) individuals had other genetic variants. Among them, 47.17% of patients presented with recurrent heart failure, while late gadolinium enhancement (LGE) was identified in 56.67% of cases that underwent magnetic resonance imaging (MRI) assessment. The variants in the A-band of *TTN* were most frequently recorded among the patients. Notably, early age-onset disease (HR = 1.008; 95% CI = 1.000–1.016; *p* = 0.037) served as a predictor of MACE in pediatrics with *TTN*-associated cardiomyopathy, and the optimal cutoff value was calculated as 75.50 months (specificity 57.1% and sensitivity 75.0%). Unfortunately, the combined genetic disorders failed to establish an association with worse outcomes in the general cohort. However, *the presence of multiple genetic variants was associated with more severe adverse outcomes* specifically in patients with dilated cardiomyopathy (DCM), with a higher prevalence of MACE occurrence.

**Conclusion:**

In our cohort, early age-onset disease was a predictor of MACE in pediatrics with *TTN*-associated cardiomyopathy. In addition, the early age of disease onset revealed a higher likelihood of MACE in the first year after diagnosis. Multiple genetic variants with *TTN* presented more severe adverse outcomes in DCM assessment.

## 1 Introduction

Titin (TTN) is the third-most abundant muscle protein in the mammalian organism, demonstrating a molecular weight of 3–4 MDa, which contributes half of the functional structure in the sarcomere (Labeit et al., 1992; Labeit et al., 1997). Its I-band segment participates in adjusting sarcomere length and force in response to cardiac stretch. This structural and functional protein is encoded by the *TTN* gene located at 2q31.2 and contains 364 exons, which are translated into several principal cardiac isoforms, according to different transcripts, including N2A, N2B, N2BA, Novex-1, Novex-2, and Novex-3 (Bang et al., 2001; Zaunbrecher et al., 2019) (Table 1). N2B isoforms contain shorter I-band regions with reduced PEVK sequences, generating higher passive tension and enhanced diastolic stiffness, and are predominantly expressed in smaller mammals, providing rapid elastic recoil. N2BA isoforms possess extended I-band regions with longer PEVK domains and additional Ig sequences, producing lower passive tension and greater extensibility and facilitating enhanced diastolic filling in larger hearts. The N2A/N2B ratio dynamically responds to hemodynamic demands, with increased N2B expression under pressure overload conditions and N2A upregulation during volume overload, representing a fundamental adaptive mechanism. The long N2BA and short N2B titin isoforms are co-expressed in the same-half of the sarcomere in large mammals’ cardiomyocytes, and the ratio between N2B and N2BA titin isoforms determines myofibrillar passive stiffness. The short N2B isoform is predominant in the left ventricle, providing stiffness support, and the long N2BA fiber demonstrates a more compliant function in maintaining cardiac diastolic movements. In addition, fetal cardiac titin, which reveals the longest transcriptional sequence, is mainly expressed in the developing embryonic heart, presents the highest compliance, and is replaced by the adult isoform after birth. Additionally, titin also contributes to skeletal muscle, and the N2A element is present in skeletal, fetal, and cardiac N2BA isoforms (Neagoe et al., 2003; LeWinter et al., 2007; Loescher et al., 2022; Tharp et al., 2019; Eldemire et al., 2021). The Novex-3 isoform, encoded by exon 48 of *TTN*, probably has no relationship with titin stiffness and structural maintenance (Bang et al., 2001). Exons 49–224 encode the central I-band region of titin, in which the N2B spring element is encoded by exon 49, the N2A element by exons 102–108, and the PEVK region by exons 109–224 (Freiburg et al., 2000).

**TABLE 1:** List of seven TTN transcripts.

Transcript	Refseq transcript	Description	Length (aa)	No. of exons
Meta	NM_00.1267550.2	Inferred complete meta-transcript	35,991	363
N2BA	NM_00.1256850.1	Principle cardiac long isoform	34,350	313
N2B	NM_003319.4	Principle cardiac long isoform	26,926	191
N2A	NM_133378.4	Soleus/skeletal long isoform	33,423	312
Novex-1	NM_133432.3	Minor cardiac short isoform	27,051	192
Novex-2	NM_133437.4	Minor cardiac short isoform	27,118	192
Novex-3	NM_133379.5	Minor cardiac short isoform	5,604	46

Titin *consists of* 34,350 amino acids and comprises four regions, with the I-band located at the N-terminal and the A-band at the C-terminal. In addition, the I-band is anchored in the Z-disk, while the A-band is bound to the thick filament (Labeit et al., 1992). The structural and functional roles of such titin segments vary and are determined by the specific amino acid sequences and domain structures in each region (Linke, 2008). The A-band titin is bound to myosin filaments and is functionally inextensible; it contains a mixture of immunoglobulin and fibronectin repeats and possesses kinase activity. The I-band titin *lies* slightly oblique between the Z-disc and the I/A-band junction and is elastic; it contains variable N2B and N2A elements, along with two regions of tandem immunoglobulin domains on either side of a PEVK region that is rich in proline, glutamate, valine, and lysine (Labeit et al., 1997; Loescher et al., 2022; Linke, 2008).

The impact of *TTN* variants depends heavily on whether the affected exon is constitutively expressed in the adult heart and at the relevant developmental stage. Titin exon characteristics are important determinants of titin-truncating variant (TTNtv) pathogenicity, which is strongly associated with dilated cardiomyopathy (DCM), hypertrophic cardiomyopathy (HCM), arrhythmic cardiomyopathy (ACM), left ventricular noncompaction cardiomyopathy (LVNC), and increased susceptibility to chemotherapy-induced cardiomyopathy. However, the missense variants are the most common type, resulting in variable clinical phenotypes. Such variants are extremely frequent in the general population, and most of them are variants of uncertain significance (VUS) (Schafer et al., 2017). Pathogenic missenses are better established in certain skeletal muscle phenotypes or specific conserved domains. The definitive links of missense variants of *TTN* to isolated cardiomyopathy are rarer and need strong segregation/functional evidence (Meyer et al., 2023; Savarese et al., 2016). Moreover, splice-altering and structural variants of *TTN* are rarely observed. A number of studies have shown that TTNtv occurs mostly in the A-band region, and children with TTNtv occurring in the A-band or M-line region often present with more severe DCM and a poorer prognosis (Akinrinade et al., 2019). However, TTNtvs are less common as a monogenic cause of pediatric-onset cardiomyopathy than in adults. In addition, biallelic or compound heterozygous TTN variants (often combining a truncation and a severe missense/in-frame change) can present with infantile cardiomyopathy or associated skeletal myopathy. Additionally, fetal/neonatal hearts express longer, more compliant titin isoforms (higher N2BA proportion). Some exons with low adult percent-spliced-in (PSI) can be more highly used in the fetal heart. Thus, a variant’s developmental expression profile can modify the pediatric risk. Accordingly, we aim to demonstrate a natural cohort of pediatric cardiomyopathy patients carrying TTN variants and attempt to establish the association between TTN-related cardiomyopathy and clinical prognosis, illustrating the risk factors and second hits in TTN variant patients that can accelerate cardiac dysfunction in the pediatric cohort.

## 2 Methods

### 2.1 Patient population

This single-center, retrospective observational study was conducted at West China Second University Hospital, Sichuan University, with patient enrollment spanning from January 2015 to December 2024. The study design adhered strictly to the Strengthening the Reporting of Observational Studies in Epidemiology (STROBE) statement guidelines to ensure methodological rigor and transparent reporting. The study protocol received approval from the Ethics Committee of West China Second University Hospital of Sichuan University (approval number: 2021-069). During hospitalization, written informed consent was obtained from all participants in accordance with institutional guidelines and international ethical standards for the potential analyses based on their clinical data and imaging records. Data collection was systematically performed by two pre-trained physicians to ensure consistency and accuracy in data acquisition. All study questionnaires underwent rigorous pretesting and subsequent revision to optimize data quality and minimize measurement error. A comprehensive quality assurance protocol was implemented, including double-verification of all questionnaires and cross-validation of clinical data through both electronic medical records and proprietary follow-up databases to ensure data integrity and completeness.

The minimum follow-up duration was established as 2 years or until death, whichever occurred first, to provide adequate observation time for the assessment of medium-term outcomes and prognostic factors. This extended follow-up period enables comprehensive evaluation of disease progression and identification of clinically relevant endpoints in the study population.

### 2.2 Inclusion and exclusion criteria

We used the following inclusion criteria to recruit candidates for further analysis: (1) all patients should meet the diagnostic criteria for DCM, HCM, ACM, or RCM, which should critically fall within the standard definition of pediatric cardiomyopathy and should be identified by echocardiography or cardiac magnetic resonance imaging (MRI), indicating significant dilation of the left ventricle with a 2SD increase compared to normal in the assessment of left ventricular end-diastolic dimension (LVEDD) or a reduced left ventricular ejection fraction (LVEF) below 50%; (2) WES should be performed, and variants of *TTN* should be identified; (3) all documented variants of *TTN* should be pathogenic, likely pathogenic, or VUS; (4) all patients should complete at least 2 years of follow-up from the initial DCM diagnosis; and (5) patients should receive the standard therapeutic strategy to maintain heart function.

The exclusion criteria included the following: (1) patients demonstrating any birth defects, especially congenital cardiovascular malformation, including coronary artery abnormality; (2) confirmed evidence demonstrating arrhythmia-associated cardiomyopathy or heart failure (HF); (3) rheumatic heart disease; (4) any cancer or tumor history; (5) previous heart surgery; (6) incomplete cardiac assessments; (7) previous diagnosis of myocarditis; (8) immune deficiency; (9) mitochondria-associated cardiomyopathy or nutritional deficiency cardiomyopathy; (10) previous infective endocarditis; and (11) genetic disorders related to other diseases.

### 2.3 Therapeutic and follow-up procedures

All patients should be identified as having left ventricular dilation by echocardiography at the beginning of the disease process and should receive standard therapeutic treatment and follow-up assessments. The therapeutic strategy for pediatric cardiomyopathy included angiotensin-converting enzyme inhibitors (ACEIs), angiotensin receptor blockers (ARBs), β-blockers, sodium–glucose cotransporter-2 inhibitor (SGLT2i), angiotensin receptor–neprilysin inhibitors (ARNis), and digoxin. All involved patients should complete at least 24 months of follow-up with continuous cardiac evaluation and therapeutic instruction. According to the study protocol, all the enrolled patients received scheduled echocardiography and ECG assessments every 6 months, while cardiac MRI was performed every 2 years. The major adverse cardiovascular events (MACEs) were considered the primary outcome and were defined as cardiac shock, heart transplantation, and cardiac device implantation. Additionally, the secondary outcome was identified as all-cause death. Accordingly, subgroups were assigned as MACE-free and MACE-attacked based on the 2 year follow-up.

### 2.4 Whole-exome sequencing analysis

The peripheral blood sample was obtained from the patient in an ethylenediaminetetraacetic acid (EDTA) anticoagulant blood sample tube and was stored at 4 °C for less than 6 h. DNA was extracted using the Blood Genome Column Medium Extraction Kit (TIANGEN Biotech, Beijing, China), according to the manufacturer’s instructions. Whole-exome sequencing (WES) was performed using the NovaSeq 6000 platform (Illumina, San Diego, CA, United States), and raw data were processed using FastP to remove adapters and filter low-quality reads. Paired-end reads were aligned to the Ensembl GRCh37/hg19 reference genome using the Burrows–Wheeler Aligner. Variant annotation was performed according to database-sourced minor allele frequencies (MAFs) and practical guidelines on pathogenicity issued by the American College of Medical Genetics. The annotation of MAFs was performed based on the 1000 Genomes, dbSNP, ESP, ExAC, and Chigene in-house MAF databases, along with Provean, Sift, PolyPhen2_hdiv, and PolyPhen2_hvar databases, using R software (R Foundation for Statistical Computing, Vienna, Austria). In our study, variants were filtered based on an MAF cutoff of <0.1% (0.001) = 0.000001 to differentiate TTN variants with possible pathogenicity versus more common variants tolerated in the general population. Variant classification was performed using a combined approach as recommended by the American College of Medical Genetics and Genomics (ACMG) 2015 guidelines.

### 2.5 Risk factor analysis

At first, the basic clinical characteristics and cardiac examination results of the enrolled patients were recorded, and all the parameters are listed in Table 1. Then, univariate analysis was performed between the *TTN*-variant-associated pediatric cardiomyopathy patients with or without MACE events during follow-up. The association was presented as a hazard ratio with a 95% CI. Then, univariate analysis was performed between patients with sole *TTN* variants and those with *TTN* variants combined with other genetic disorders. In addition, all significant associated parameters were used to build receiver operating characteristic (ROC) curves to evaluate the risk for MACE in *TTN-*variant-related cardiomyopathy.

### 2.6 Statistical analysis

IBM SPSS Statistics 27.0.1(SPSS, Chicago, United States) was used for the statistical analysis. Continuous data with a normal distribution were presented as the means ± standard deviations, and the clinical characteristics of each group were compared using a two-tailed, unpaired t-test, while skewed distributions were presented as medians with quartiles and analyzed using the Mann–Whitney U-test. Categorical data were described as n (%), and cross-group analyses were performed using the chi-square test or Fisher’s exact test. A two-sided *p*-value < 0.05 indicated statistical significance. Analysis was performed in R version 3.4.3 (R Foundation for Statistical Computing, Vienna, Austria) using the meta, survival, and ROC packages.

## 3 Results

### 3.1 Clinical characteristics of study subjects

From January 2015 to December 2024, 220 patients in this cohort underwent WES and were diagnosed with cardiac disease without a definite etiology. Among those tested, 53 were found to carry TTN variants, representing 24.1% (53/220) of the genetically tested patients. The 53 patients revealed TTN variants with a mean age of 54.7 ± 54.3 months. The baseline characteristics of the cohort are summarized in Supplementary Table S1. The presenting symptoms were complex in the cohort with DCM, HCM, RCM, ACM, LVNC, and EFE. The number of patients in each cardiac function class, according to the New York Heart Association (NYHA) or ROSS classification, was 15 (28.30%), 10 (18.87%), 18 (33.96%), and 10 (18.87%), respectively. The average values of cTnT and BNP of enrolled patients were 46.31 ± 281.19 ug/L and 4,962.89 ± 3,853.98 pg/mL, respectively. Heart failure with reduced ejection fraction (HFrEF) was observed in 25 (47.17%) patients, and the average LVEDD was 45.09 ± 10.27 mm among 53 individuals. A total of 30 of 53 patients underwent cardiac MRI, with 17 of them being late gadolinium enhancement (LGE)-positive. Twenty-one (39.62%) patients were recorded as having significant arrhythmias, including atrioventricular block (AVB, 20.75%), atrial tachycardia (AT, 5.67%), and ventricular tachycardia (VT, 15.09%), on initial Holter or ECG recordings (Table 2). Forty-eight of fifty-three (90.50%) individuals carried genetic variants on other genes (Supplementary Table S2), which were considered non-major contributing pathogenic variants to the presented cardiomyopathy. Among the enrolled patients, DCM manifestation compromised the majority of the population with *TTN*-variant-associated cardiomyopathy.

**TABLE 2:** Comparison of clinical characteristics of 55 subjects in the cohort.

Characteristic	Isolated TTN variants (n = 9)	Compound variants in multiple genes (n = 46)	Sig.
Female	6 (66.67)	17 (36.96)	​
Male (%)	3 (33.33)	29 (63.04)	​
Age (months)	17 (9.50–74.50)	26 (4.70–100.00)	0.856
Age at diagnosis (month)	20 (12.06–77.00)	52.5 (12.30,110.00)	0.473
Positive family history	0 (0.00)	2 (4.35)	1.000
Identified myocardial injuries	4 (44.44)	29 (63.04)	<0.001*
LVEF	61 (24–69)	53 (32–71)	0.360
Malignant arrhythmia	​	​	1.000
VT	1 (11.11)	6 (13.04)	​
VF	0 (0.00)	1 (2.17)	​
AF	0 (0.00)	1 (2.17)	​
CAVB	0 (0.00)	3 (6.52)	​
ICD implantation	0 (0.00)	3 (6.52)	1.000
Death	0 (0.00)	2 (4.35)	1.000

LVEF, left ventricular ejection fraction; ICD, implantable cardioverter defibrillator; VT, ventricular tachycardia; VF, ventricular fibrillation; AF, atrial flutter.

### 3.2 Prognosis of *TTN*-variant-associated cardiomyopathy

**FIGURE 1:**
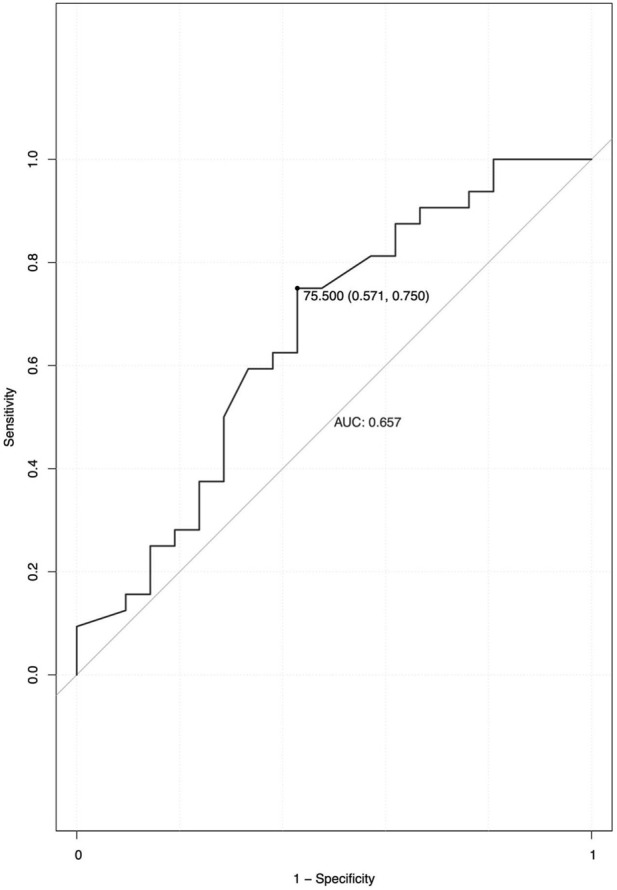
Receiver operating characteristic analysis of age onset cardiac disease for predicting MACE.

**FIGURE 2:**
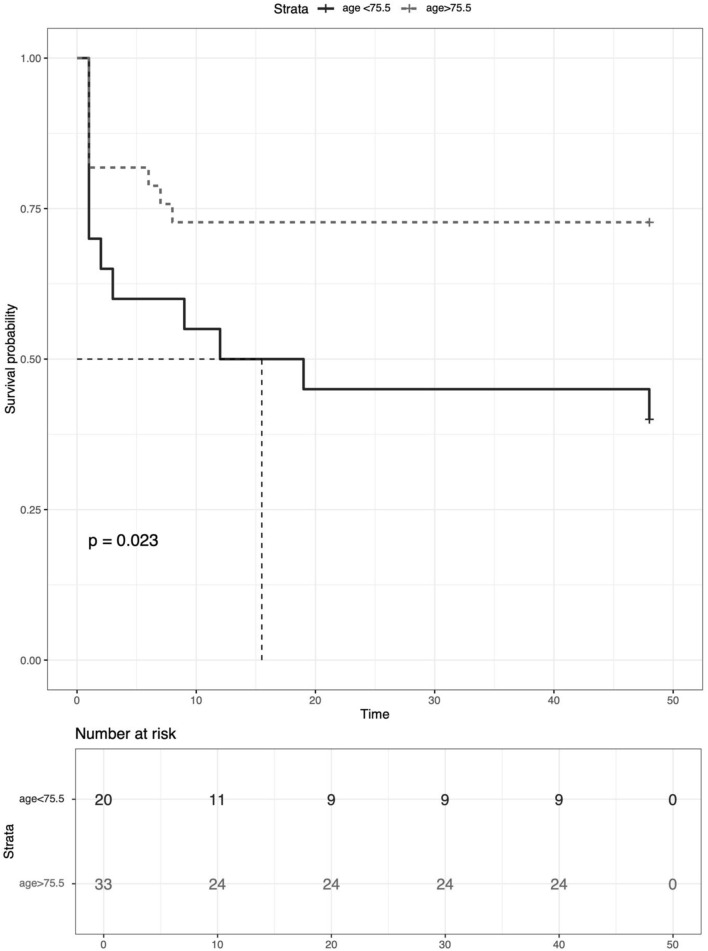
Kaplan–Meier estimates of time to MACE for patients with TTN mutations in our cohort between onset cardiomyopathy earlier than 75.5 months and later than 75.5 months.

Twenty-three of fifty-three (43.40%) patients experienced MACE during at least 48 months of follow-up. Six of them died, one underwent heart transplantation, and one received a cardiac pacemaker. Univariable baseline predictors of the endpoint in *TTN*-associated cardiomyopathy are shown in Table 3. Univariable Cox regression incorporating 53 patients (23 events) demonstrated that early age-onset disease (HR = 1.008, 95% CI = 1.000–1.016; *p* = 0.037) was a predictor of the MACE in pediatrics with *TTN*-associated cardiomyopathy (Table 4). In ROC analysis, the area under the curve for age-onset cardiac disease in predicting MACE was 0.657 (Figure 1). The best cutoff value for age at initial disease onset was 75.5 months, yielding a specificity and a sensitivity of 57.1% and 75.0%, respectively, and demonstrating that the MACE mainly occurred approximately 1 year after disease onset, most likely within 10 months in early age-onset patients (45%), while the likelihood of MACE in late-onset patients was reduced to 27.2% (Figure 2). A total of 24 subjects experienced a severe decrease in LVEF (<40%), mainly among early-onset patients (85.7%).

**TABLE 3:** Univariate analysis of risk factors between sole TTN variants and multiple variant-associated cardiomyopathy.

Characteristic	Group A (n = 5)	Group B (n = 48)	Sig.
Sex			
Female (%)	3 (60.0)	20 (41.7)	0.642
Male (%)	2 (40.0)	28 (58.3)	​
Age (months)	20 (15.0–153.0)	26 (0.3–166.0)	0.895
MACE (%)	2 (40.0)	19 (39.6)	1.000
cTnT (ug/L)	0.081 (0.012–0.976)	0.165 (0.006–1734)	0.734
BNP (pg/mL)	2,145.3 (1788.6–2,293.2)	1,438.9 (12.3–35,000)	0.541
LVEF			
≤40%	4 (80.0)	24 (50.0)	0.355
>40%	1 (20.0)	24 (50.0)	​
DCM (%)	4 (80.0)	21 (43.8)	0.380
Arrhythmias (%)	2 (40.0)	19 (39.6)	1.000
NYHA or ROSS (%)			
I–II	2 (40.0)	26 (54.2)	0.658
III–IV	3 (60.0)	22 (45.8)	​
TTN A (%)	3 (60.0)	27 (56.3)	1.000
Type of titin isoforms			
N2A	1 (20.0)	9 (18.8)	1.000
Novex-3	0 (00.0)	2 (4.2)	0.645
Full-length	4 (80.0)	37 (77.0)	1.000

**TABLE 4:** The univariable analysis of risk factor of TTN-cardiac disease.

Characteristic	N	*P* value	Hazard ratio (95% CI)
Age	53	0.037*	1.008 [1.000, 1.016]
cTnT	38	0.096	1.001 [1.000, 1.002]
BNP	39	0.099	1.000 [1.000, 1.000]
LVEF	53	0.826	1.002 [0.981, 1.024]
RA	31	0.118	1.044 [0.989, 1.101]
RV	31	0.228	1.035 [0.979, 1.095]
LA	39	0.912	0.997 [0.939, 1.058]
LV	40	0.520	1.017 [0.967, 1.069]
TTN A-band	​	​	​
Yes	30	​	​
No	23	0.864	1.078 [0.454, 2.560]
DCM	​	​	​
Yes	25	​	​
No	28	0.377	1.540 [0.638, 3.718]
Male	​	​	​
Yes	30	​	​
No	23	0.317	0.629 [0.254, 1.559]
NYHA	​	​	​
I–II	28	​	​
III–IV	25	0.668	0.668 [0.277, 1.614]
Arrhythmia	​	​	​
Yes	21	​	​
No	32	0.822	0.905 [0.381, 2.149]
LVEF ≤40%	​	​	​
Yes	28	​	​
No	25	0.950	1.028 [0.436, 2.421]

### 3.3 Additional genetic variants contributed to severe outcomes in *TTN*-associated DCM

**FIGURE 3:**
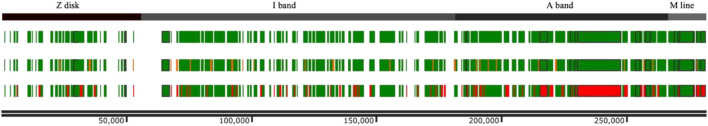
Spatial distribution of TTN mutations. In the first line, green vertical line arrows represent TTN exons; in the second line, orange represents the mutations in our cohort that present DCM; in the third line, red represents the mutations in previous articles, which present DCM; black and gray rectangular frames represent Z-disk, I-band, A-band, and M-line.

According to the results of WES analysis, *TTN* variants were distributed among exons 3–358, affecting the N2A titin isoform (NM_133378.4), the Novex-3 titin isoform (NM_133379.5), and the full-length TTN (NM_001267550.2). Previous studies reported that *TTN* variants were mainly located in the A-band. Consequently, most *TTN* variants were also identified within the domain of the A-band (56.60%) in our cohort; meanwhile, the percentages of patients with *TTN* variants located in the I-band, M-band, and Z-disk coding sequences were 54.72%, 18.87%, and 32.08%, respectively (Table 2; Figure 3). Then, the identified *TTN* variants associated with DCM in this cohort were mapped into previously reported variants (Figure 3), and the results demonstrated that the pediatric patients failed to reveal any fundamental variants, while the *TTN* variants in adult patients were mainly observed in the A-band coding sequence.

Moreover, we established two subgroups to evaluate the differences between sole *TTN* variants (group A) and *TTN* combined with other genetic disorder (group B)-associated cardiomyopathy (Table 3). In group A, one subject was identified with a variant associated with the N2A titin isoform, while in group B, nine patients had variants involving the N2A titin isoform, and two cases displayed variants related to the Novex-3 titin isoform. However, the combined variants with *TTN* variants were unable to present a higher incidence of MACE attacks. There were three types of gene variants, namely, base substitution (162/169, 95.86%), deletion (6/169, 3.55%), and duplication (1/169, 0.59%), and base substitution was the most common variant in such cohorts. In addition, the gene variants in our cohort were classified into four pathogenicity categories, namely, LB (likely benign) (1/169, 0.59%), VUS (variant of uncertain significance) (153/169, 90.53%), LP (likely pathogenic) (12/169, 7.10%), and P (pathogenic) (3/169, 1.78%) (Supplementary Table S2). Thus, in general, cases combined with no definitely pathogenic or likely pathogenic variants did not demonstrate an aggressive adverse prognosis among all types of cardiomyopathy patients. However, the additional genetic variants contributed to secondary assessment outcomes: one patient received cardiac resynchronization therapy, one underwent heart transplantation, and six lethal events were recorded among *TTN*-associated cardiomyopathy patients with combined variants. Base substitutions in full-length TTN accounted for the majority of all variants. However, the type of variants did not appear to affect cardiac function in *TTN*-associated cardiomyopathy (Table 3).

## 4 Discussion

*TTN*, encoding the largest human protein spanning 364 exons across 281 kb, demonstrates significant genetic heterogeneity across multiple cardiomyopathy phenotypes due to its extensive coding sequence and complex alternative splicing. In DCM, TTNtvs represent the most prevalent genetic cause, accounting for 15%–25% of cases through haploinsufficiency mechanisms that compromise sarcomere stability and elastic properties, particularly when located in the A-band region. HCM is associated with *TTN* missense variants affecting titin–myosin interactions and calcium sensitivity, typically presenting with milder hypertrophy and late-onset phenotypes compared to other sarcomeric gene mutations. RCM primarily involves I-band region mutations, especially in the PEVK domain, leading to increased myocardial stiffness and impaired diastolic filling while preserving systolic function. LVNC demonstrates TTN-truncating variants similar to DCM, but with distinct morphological features involving impaired ventricular wall compaction during embryogenesis. ACM shows rare *TTN* variants contributing to sarcomere destabilization, fibrofatty replacement, and arrhythmogenic substrate development. Genotype–phenotype correlations reveal that A-band variants demonstrate stronger disease associations, while variant location, genetic background, and sex-specific factors influence penetrance and clinical severity. These findings underscore TTN’s critical role in cardiac structure and function, with significant implications for precision medicine approaches, family screening strategies, and risk stratification in cardiomyopathy management.

*TTN* truncation variants have been associated with cardiomyopathies or arrhythmias and neuromuscular disease. However, many studies have focused on genetic variation in DCM-related genes in adult populations; the mutational landscape in pediatric DCM patients remains undetermined. We reviewed previous studies and found that the prevalence of *TTN* truncation variants in DCM and HCM was 10% and 3%, respectively, which is consistent with previous reports (Vatta et al., 2025; Ware and Cook, 2018). However, the heterogeneity of the meta-analysis exceeded 75%, which may be due to some TTN mutations being ignored or overlooked. Therefore, clarifying the clinical phenotype and prognosis of *TTN*-variant-associated cardiac disease is important for more objective assessment and improved patient management, especially in pediatric cases.

In this retrospective cohort study, we use WES in this context to provide a standardized comparison between groups. We found that the ages of cardiomyopathy development in patients with sole *TTN* variants and those with multiple variants were both approximately 2 years, and multiple variants were associated with a more adverse outcome among DCM patients. We found that early-onset disease was an independent risk factor for MACE in *TTN*-variant-associated cardiomyopathy in pediatric patients. Thus, the promoted and enhanced therapeutic strategy should be considered for such patients.

DCM had the largest majority in both groups, and among them, 80.0% (20/25) subjects had an LVEF of less than 40%. DCM is the most common type of cardiomyopathy in children, characterized by the presence of left ventricular dilatation and global or regional systolic dysfunction unexplained solely by abnormal loading conditions that can lead to heart failure and sudden cardiac death in children (Arbelo et al., 2023; Jammal Addin et al., 2019). Titin, also known as connectin, is a giant sarcomere protein found in both cardiac and skeletal muscle (Labeit et al., 1997; Bang et al., 2001). Titin is essential to regulate myocardial function, including heart filling and pumping blood (Loescher et al., 2022). *TTN* variants are a common cause of DCM genetic etiology, and *TTN*-truncating variants are the main contributor, occurring in up to 15% of all DCM patients (Ware and Cook, 2018). A recent study characterizing the genetic basis of DCM heart transplantation in the Chinese population found that *TTN*-truncating variants accounted for 18.8% of their cohort (Lian et al., 2023). Another study recently reported that the detection rate of the DCM gene mutation in children was 39% in their cohort, which is consistent with previous studies to a certain extent (Wang et al., 2022), and suggested that the prognosis of children with DCM caused by the pathogenic gene mutation was worse (Zheng et al., 2023). In our study, 24 subjects had an LVEF of less than 40%, mainly from group B (85.7%). In the group with LVEF less than 40%, there were 20 children with DCM, which accounted for 80.0% (20/25) of those diagnosed with DCM and 84.0% (21/25) of those with other genetic mutations. This indicated that, compared with TTN mutations alone, children with TTN mutations incorporating other gene mutations showed a trend toward worse outcomes.

In our study, *TTN* variants were distributed in exons 3–358, including Z-disk, I-band, and A-band regions, and involved two titin isoforms—N2A titin isoform and Novex-3 titin isoform. The N2A region *consists of* four immunoglobulin domains and an insertion sequence, binding F-actin in a calcium-dependent manner (Tsiros et al., 2022). The Novex-3 isoform may play a role as a structural and regulatory element (Bang et al., 2001). In this cohort, there were two subjects involving the Novex-3 titin isoform—one of them was DCM. The patients who suffered from DCM not only had TTN mutations but also *FLNC* and *BMPR2* mutations and had a poor prognosis. FLNC encodes filamin C, an important Z-disk protein located at the Z-disks, costameres, and intercalated disks (Brodehl et al., 2017). Based on ClinGen gene validation efforts, *FLNC* pathogenic variants have definite contributions to DCM. DCM has genetic and allelic heterogeneity—many different variants in many different genes can cause the same phenotype. In our cohort, DCM subjects possessed different pathogenic gene mutations such as *MYH7*, *TNNT2*, and *RBM20*.

A number of studies have shown that TTNtv occurs mostly in the A-band region, while a small proportion of TTNtv occurs in the I-band, Z-disk, and M-line region, and children with TTNtv occurring in the A-band or M-line region often present with more severe DCM and a poor prognosis (Akinrinade et al., 2019; Romano et al., 2022). Previous studies reported that TTN mutations were mainly located in the A-band, which was the same as in our cohort. The A-band is the longest region of TTN, which comprises 49.8% of the TTN coding region and is composed of regular patterns of immunoglobulin (Ig)-like and fibronectin type 3 repeats (LeWinter et al., 2007; Loescher et al., 2022). A-band enrichment of TTNtv in patients with DCM has been reported several times, and it is probably associated with exon length ratio and asymmetric exons, for which the translation reading frame may change and make it more likely to produce abnormal or truncated titin protein (Schafer et al., 2017; Haggerty et al., 2019; Kim et al., 2022; Rich et al., 2020). However, there were no signification differences in TTN-variant location with MACE in our pediatric cohort. Therefore, we indicate that the spatial distribution of TTN mutations had no significant difference in cardiac function, but children *tended to* have a poor prognosis with DCM caused by TTN mutations incorporating other gene mutations.

This study has some limitations. First, it was a retrospective, single-center study, with a small sample size. In addition, it was a preliminary exploration of pediatric TTN-associated cardiomyopathy, which provides a basis for subsequent large-sample studies. Therefore, it is necessary to establish multi-center, large-sample research to better explain the relationship between the spatial distribution of *TTN* variants and clinical outcomes of the disease.

## Data Availability

The original contributions presented in the study are publicly available. This data can be found in The National Genomics Data Center (NGDC) at https://ngdc.cncb.ac.cn with the accession number PRJCA060523.
