# Supplementary material for: TTN variants in pediatric cardiomyopathy: a retrospective cohort study
Source: Front Genet. 2026 Apr 1;17:1758524. doi: 10.3389/fgene.2026.1758524 (PMC13078735; doi:10.3389/fgene.2026.1758524)
Supplement: Supplementary file 1 [file Table1.docx]

| Variable  Supplementary Table1. Baseline Characteristics (n=53) | Mean±SD or n(%) |
| --- | --- |
| Age onset diseases, months | 54.7±54.3 |
| NYHA or Ross |  |
| I | 15(28.30) |
| II | 10(18.87) |
| III | 18(33.96) |
| IV | 10(18.87) |
| Cardiac disease phenotype |  |
| DCM | 25(47.17) |
| HCM | 4(7.55) |
| RCM | 3(5.66) |
| ACM | 2(3.77) |
| EFE | 3(5.66) |
| LVNC | 5(9.43) |
| PAH | 5(9.43) |
| AVB | 3(5.66) |
| VT | 3(5.66) |
| cTnT, ug/L | 46.31±281.19 |
| BNP, pg/mL | 4962.89±3853.98 |
| LVEF |  |
| LVEF≥50% | 26(49.06) |
| 40%<LVEF <50% | 2(3.77) |
| LVEF≤40% | 25(47.17) |
| LVED, mm | 45.09±10.27 |
| LGE | 17(56.67) |
| AVB |  |
| I | 7(13.21) |
| II | 1(1.87) |
| III | 3(5.67) |
| AT | 3(5.67) |
| VT | 8(15.09) |
| MACE |  |
| Shock | 15(28.30) |
| Death | 6(11.32) |
| Heart transplantation | 1(1.89) |
| Cardiac pacemaker | 1(1.89) |
| With other gene mutation | 48(90.50) |
| TTN domain |  |
| A band | 30(56.60) |
| I band | 29(54.72) |
| M band | 10(18.87) |
| Z disk | 17(32.08) |
