# Supplementary material for: TTN variants in pediatric cardiomyopathy: a retrospective cohort study
Source: Front Genet. 2026 Apr 1;17:1758524. doi: 10.3389/fgene.2026.1758524 (PMC13078735; doi:10.3389/fgene.2026.1758524)
Supplement: Supplementary file 2 [file Table2.docx]

Supplementary Table 2.The TTN variants and a full list of the variants in other genes identified in each patient.

| *Patient* | *Gene* | *Refseq transcript* | *Coding change* | *Protein change* | *Type of Variant* | *AGMG variant rating* | *GnomAD MAF* |
| --- | --- | --- | --- | --- | --- | --- | --- |
| *NO.1* | *TTN* | *NM_001267550.2* | *exon316: c.66590G>A* | *p.Arg22197Gln* | *base substitution* | *VUS* | *0.000737212* |
|  | *TTN* | *NM_001267550.2* | *exon232: c.42850C>T* | *p.Arg14284Cys* | *base substitution* | *VUS* | *0.0000161093* |
|  | *MYH7* | *NM_000257.4* | *exon23: c.2788G>A* | *p.Glu930Lys* | *base substitution* | *LP* | *--* |
| *NO.2* | *TTN* | *NM_001267550.2* | *exon358: c.103859G>A* | *p.Arg34620His* | *base substitution* | *VUS* | *0.000222841* |
|  | *TTN* | *NM_001267550.2* | *exon324: c.68986A>G* | *p.Ile22996Val* | *base substitution* | *VUS* | *--* |
|  | *TTN* | *NM_001267550.2* | *exon41: c.9497T>C* | *p.Val3166Ala* | *base substitution* | *VUS* | *--* |
|  | *CAPN3* | *NM_000070.3* | *exon20: c.2120A>G* | *p.Asp707Gly* | *base substitution* | *P* | *0.00206589* |
| *NO.3* | *TTN* | *NM_001267550.2* | *exon325: c.69586G>A* | *p.Asp23196Asn* | *base substitution* | *VUS* | *0.0000080406* |
|  | *TTN* | *NM_001267550.2* | *exon114: c.30803-22A>T* |  | *base substitution* | *VUS* | *--* |
|  | *TTN* | *NM_001267550.2* | *exon48: c.14074C>G* | *p.Leu4692Val* | *base substitution* | *VUS* | *--* |
| *NO.4* | *TTN* | *NM_001267550.2* | *exon358: c.104024G>A* | *p.Arg34675Lys* | *base substitution* | *VUS* | *0.0002* |
|  | *TTN* | *NM_001267550.2* | *exon18: c.2996G>A* | *p.Arg999His* | *base substitution* | *VUS* | *0.0003* |
|  | *FHOD3* | *NM_001281740.3* | *exon27: c.4570G>A* | *p.Val1524Met* | *base substitution* | *VUS* | *0.00001213* |
|  | *RYR2* | *NM_001035.3* | *exon50: c.7552C>T* | *p.Arg2518Trp* | *base substitution* | *VUS* | *0.00004012* |
| *NO.5* | *TTN* | *NM_001267550.2* | *exon238: c.44078G>A* | *p.Arg14693His* | *base substitution* | *VUS* | *0.0006* |
|  | *TTN* | *NM_001267550.2* | *exon206: c.39418G>A* | *p.Ala13140Thr* | *base substitution* | *VUS* | *0.0003* |
|  | *DSG2* | *NM_001943.5* | *exon7: c.706A>G* | *p.Thr236Ala* | *base substitution* | *VUS* | *0.0013* |
|  | *PRKAG2* | *NM_016203.4* | *exon2: c.130G>A* | *p.Ala44Thr* | *base substitution* | *VUS* | *0.0017* |
| *NO.6* | *TTN* | *NM_001267550.2* | *exon326:c.83596_83600del* | *p.Pro27866Trpfs*7* | *deletion* | *LP* | *--* |
|  | *TTN* | *NM_001267550.2* | *exon256: c.48103C>A* | *p.Leu16035Met* | *base substitution* | *VUS* | *--* |
|  | *TTN* | *NM_001267550.2* | *exon225:c.41179C>T* | *p.Arg13727Cys* | *base substitution* | *VUS* | *0.0000139712* |
| *NO.7* | *TTN* | *NM_001267550.2* | *exon358: c.105311T>C* | *p.Met35104Thr* | *base substitution* | *VUS* | *0.00000698519* |
|  | *TTN* | *NM_001267550.2* | *exon66: c.19282A>T* | *p.Ser6428Cys* | *base substitution* | *VUS* | *0.00000698061* |
|  | *MYH6* | *NM_002471.4* | *exon31: c.4369G>T* | *p.Glu1457** | *base substitution* | *VUS* | *0.00005445* |
|  | *PRDM16* | *NM_022114.4* | *exon9: c.1759T>C* | *p.Cys587Arg* | *base substitution* | *VUS* | *0.000004015* |
| *NO.8* | *TTN* | *NM_001267550.2* | *exon46: c.11254+1G>C* |  | *base substitution* | *LP* | *0.00011333* |
|  | *TTN* | *NM_001267550.2* | *exon102: c.29168G>A* | *p.Gly9723Asp* | *base substitution* | *VUS* | *0.00000403* |
|  | *VCL* | *NM_014000.3* | *exon1: c.32G>A* | *p.Ser11Asn* | *base substitution* | *VUS* | *0.0005* |
|  | *FLNA* | *NM_001110556.2* | *exon10: c.1510A>G* | *p.Lys504Glu* | *base substitution* | *VUS* | *--* |
| *NO.9* | *TTN* | *NM_001267550.2* | *exon335: c.90370G>T* | *p.Glu30124** | *base substitution* | *P* | *--* |
|  | *LRPPRC* | *NM_133259.4* | *exon14: c.1621A>G* | *p.Arg541Gly* | *base substitution* | *VUS* | *0.00005438* |
| *NO.10* | *TTN* | *NM_001267550.2* | *exon358: c.105490C>T* | *p.Arg35164Cys* | *base substitution* | *VUS* | *0.0001134* |
|  | *MYPN* | *NM_032578.4* | *exon12: c.2565-4A>G* |  | *base substitution* | *VUS* | *0.00001194* |
|  | *PPP1R13L* | *NM_006663.4* | *exon11: c.2086C>A* | *p.Pro696Thr* | *base substitution* | *VUS* | *--* |
| *NO.11* | *TTN* | *NM_001267550.2* | *exon125: c.32012-10G>A* |  | *base substitution* | *VUS* | *0.000004271* |
|  | *TTN* | *NM_001267550.2* | *exon358: c.103412G>A* | *p.Arg34471Gln* | *base substitution* | *VUS* | *0.0035* |
|  | *GAA* | *NM_000152.5* | *exon13: c.1827C>G* | *p.Tyr609** | *base substitution* | *LP* | *--* |
|  | *GAA* | *NM_000152.5* | *exon14: c.1889-53_1932del* |  | *deletion* | *LP* | *--* |
|  | *TNNI3* | *NM_000363.5* | *:exon3: c.37C>T* | *p.Arg13Cys* | *base substitution* | *VUS* | *--* |
|  | *BMPR2* | *NM_001204.7* | *exon12: c.2804C>T* | *p.Ala935Val* | *base substitution* | *VUS* | *0.000007959* |
|  | *SYNE1* | *NM_182961.4* | *exon107: c.19772A>G* | *p.Tyr6591Cys* | *base substitution* | *VUS* | *0.0000139616* |
|  | *SCN5A* | *NM_000335.5* | *exon17: c.2893C>T* | *p.Arg965Cys* | *base substitution* | *VUS* | *0.0006* |
| *NO.12* | *TTN* | *NM_133378.4* | *exon18: c.2996G>A* | *p.Arg999His* | *base substitution* | *VUS* | *0.0003* |
|  | *TTN* | *NM_133378.4* | *exon307: c.96320G>A* | *p.Arg32107Lys* | *base substitution* | *VUS* | *0.0002* |
|  | *TTN* | *NM_133378.4* | *exon60: c.14675G>A* | *p.Arg4892Gln* | *base substitution* | *VUS* | *0.0055* |
|  | *TNNT2* | *NM_001001430.3* | *exon13: c.629_631del* | *p.Lys210del* | *deletion* | *P* | *--* |
| *NO.13* | *TTN* | *NM_001267550.2* | *exon75: c.21683-27T>C* |  | *base substitution* | *VUS* | *0.0004* |
|  | *TTN* | *NM_001267550.2* | *exon48: c.11663G>T* | *p.Gly3888Val* | *base substitution* | *VUS* | *--* |
|  | *RBM20* | *NM_001134363.3* | *exon2: c.1177C>A* | *p.Pro393Thr* | *base substitution* | *VUS* | *0* |
| *NO.14* | *TTN* | *NM_133378.4* | *exon34: c.8069C>T* | *p.Thr2690Ile* | *base substitution* | *VUS* | *0.0073* |
|  | *ACTN2* | *NM_001103.4* | *exon1: c.55T>G* | *p.Tyr19Asp* | *base substitution* | *VUS* | *--* |
| *NO.15* | *TTN* | *NM_001267550.2* | *exon198: c.38755G>A* | *p.Ala12919Thr* | *base substitution* | *VUS* | *0.0004* |
|  | *RYR2* | *NM_001035.3* | *exon3: c.256G>A* | *p.Val86Met* | *base substitution* | *VUS* | *0.00005562* |
|  | *AKAP9* | *NM_005751.5* | *exon38: c.9257A>G* | *p.Asp3086Gly* | *base substitution* | *VUS* | *--* |
| *NO.16* | *TTN* | *NM_133378.4* | *exon28: c.5255G>A* | *p.Arg1752His* | *base substitution* | *VUS* | *0.0002* |
|  | *KMT2D* | *NM_003482.4* | *exon4: c.271dupT* | *p.Trp91Leufs*17* | *duplication* | *LP* | *--* |
|  | *CSRP3* | *NM_003476.5* | *exon4: c.371G>T* | *p.Gly124Val* | *base substitution* | *VUS* | *--* |
| *NO.17* | *TTN* | *NM_133378.4* | *exon148: c.30884-10A>C* |  | *base substitution* | *VUS* | *0.0002* |
|  | *ACTC1* | *NM_005159.5* | *exon5: c.635G>A* | *p.Arg212His* | *base substitution* | *VUS* | *--* |
|  | *JPH2* | *NM_020433.5* | *exon3: c.1267G>T* | *p.Ala423Ser* | *base substitution* | *VUS* | *0.0008* |
| *NO.18* | *TTN* | *NM_001267550.2* | *exon204: c.39295G>A* | *p.Val13099Met* | *base substitution* | *VUS* | *0.00000699* |
| *NO.19* | *TTN* | *NM_001267550.2* | *exon34: c.8069C>T* | *p.Thr2690Ile* | *base substitution* | *VUS* | *0.0073* |
|  | *TNNI3* | *NM_000363.5* | *exon5: c.235C>T* | *p.Arg79Cys* | *base substitution* | *VUS* | *0.00608855* |
|  | *AKAP9* | *NM_005751.5* | *exon9: c.3430T>C* | *p.Cys1144Arg* | *base substitution* | *VUS* | *0.0045* |
|  | *MYBPC3* | *NM_000256.3* | *exon12: c.1000G>A* | *p.Glu334Lys* | *base substitution* | *VUS* | *0.0034* |
| *NO.20* | *TTN* | *NM_133379.5* | *exon46: c.14113C>T* | *p.Arg4705** | *base substitution* | *VUS* | *0.0003* |
|  | *FLNC* | *NM_001458.5* | *exon40: c.6632C>G* | *p.Thr2211Ser* | *base substitution* | *VUS* | *--* |
|  | *BMPR2* | *NM_001204.7* | *exon8: c.1042G>A* | *p.Val348Ile* | *base substitution* | *VUS* | *0.0074* |
| *NO.21* | *TTN* | *NM_001267550.2* | *exon280: c.54148C>T* | *p.Arg18050Cys* | *base substitution* | *VUS* | *0.0079* |
|  | *AKAP9* | *NM_005751.5* | *exon46: c.11234G>A* | *p.Gly3745Glu* | *base substitution* | *VUS* | *0.0000418696* |
|  | *ANK2* | *NM_001148.6* | *exon9: c.881C>T* | *p.Ala294Val* | *base substitution* | *VUS* | *--* |
| *NO.22* | *TTN* | *NM_133378.4* | *exon300:c.90350A>G* | *p.Glu30117Gly* | *base substitution* | *VUS* | *--* |
|  | *TTN* | *NM_133378.4* | *exon254:c.55648C>T* | *p.Arg18550Trp* | *base substitution* | *VUS* | *--* |
|  | *TTN* | *NM_133378.4* | *exon207:c.40649A>G* | *p.Asp13550Gly* | *base substitution* | *VUS* | *--* |
|  | *TTN* | *NM_133378.4* | *exon76:c.19297G>A* | *p.Gly6433Arg* | *base substitution* | *VUS* | *--* |
| *NO.23* | *TTN* | *NM_001267550.2* | *exon129:c.32443A>G* | *p.Lys10815Glu* | *base substitution* | *VUS* | *0.0000262795* |
|  | *TTN* | *NM_001267550.2* | *exon328:c.87263C>T* | *p.Thr29088Ile* | *base substitution* | *VUS* | *0.000334374* |
|  | *TTN* | *NM_001267550.2* | *exon326:c.79865C>T* | *p.Pro26622Leu* | *base substitution* | *VUS* | *0.000334225* |
|  | *MYH7* | *NM_000257.4* | *exon9:c.771G>T* | *p.Lys257Asn* | *base substitution* | *VUS* | *--* |
| *NO.24* | *TTN* | *NM_001267550.2* | *exon54:c.16028C>T* | *p.Ser5343Phe* | *base substitution* | *VUS* | *0.0002* |
| *NO.25* | *TTN* | *NM_001267550.2* | *exon63:c.18407G>A* | *p.Arg6136Gln* | *base substitution* | *LP* | *--* |
|  | *MYL2* | *NM_000432.4* | *exon3:c.106A>G* | *p.Met36Val* | *base substitution* | *VUS* | *--* |
| *NO.26* | *TTN* | *NM_001267550.2* | *exon305:c.63352C>T* | *p.Arg21118Trp* | *base substitution* | *LP* | *0.0017* |
|  | *TTN* | *NM_001267550.2* | *exon28:c.5231C>T* | *p.Pro1744Leu* | *base substitution* | *VUS* | *--* |
|  | *TCAP* | *NM_003673* | *exon2:c.428T>G* | *p.Val143Gly* | *base substitution* | *LP* | *--* |
|  | *SYNE2* | *NM_182914.3* | *exon103:c.18623G>T* | *p.Arg6208Leu* | *base substitution* | *LP* | *--* |
|  | *SYNE2* | *NM_182914.3* | *exon107:c.19335T>C* | *p.Ala6445Asp* | *base substitution* | *VUS* | *0.00024* |
| *NO.27* | *TTN* | *NM_001267550.2* | *exon129:c.C32462T* | *p.Pro10821Leu* | *base substitution* | *VUS* | *--* |
|  | *FLNC* | *NM_001458* | *exon46:c.7562G>A* | *p.Gly2521Asp* | *base substitution* | *VUS* | *--* |
|  | *HCN4* | *NM_005477* | *exon8:c.3064C>T* | *p.Arg1022X, 182* | *base substitution* | *VUS* | *--* |
| *NO.28* | *TTN* | *NM_001267550.2* | *exon326:c.73517G>A* | *p.Gly24506Asp* | *base substitution* | *VUS* | *0* |
|  | *TTN* | *NM_001267550.2* | *exon34:c.8069C>T* | *p.Thr2690Ile* | *base substitution* | *VUS* | *0.002* |
|  | *TTN* | *NM_001267550.2* | *exon266:c.50187T>A* | *p.Asn16729Lys* | *base substitution* | *VUS* | *--* |
|  | *MYBPC3* | *NM_000256.3* | *exon27:c.2761C>G* | *p.Gln921Glu* | *base substitution* | *VUS* | *0.001* |
|  | *RYR2* | *NM_001035.3* | *exon38:c.5774T>C* | *p.Ile1925Thr* | *base substitution* | *LP* | *--* |
| *NO.29* | *TTN* | *NM_001267550.2* | *exon34:c.C8069T* | *p.Thr2690Ile* | *base substitution* | *VUS* | *0.0002106* |
|  | *ACTN2* | *NM_001278344.2* | *exon10:c.91delC* | *p.Pro31Phe* | *deletion* | *VUS* | *--* |
|  | *RYR2* | *NM_001035.3* | *exon38:c.T5774C* | *p.Ile1925Thr* | *base substitution* | *VUS* | *0.00003532* |
| *NO.30* | *TTN* | *NM_001267550.2* | *exon129:c.C32462T* | *p.Pro10821Leu* | *base substitution* | *VUS* | *0.001169* |
|  | *VCL* | *NM_014000.3* | *exon17:c.G2521C* | *p.Asp841His* | *base substitution* | *VUS* | *0.0003407* |
|  | *JUP* | *NM_002230.4* | *exon4:c.C560T* | *p.Ala187Val* | *base substitution* | *VUS* | *0* |
| *NO.31* | *TTN* | *NM_001267550.2* | *exon129:c.C32462T* | *p.Pro10821Leu* | *base substitution* | *VUS* | *0.001169* |
|  | *TTN* | *NM_001267550.2* | *exon272:c.T51459G* | *p.Asp17153Glu* | *base substitution* | *VUS* | *--* |
|  | *SCN5A* | *NM_000335.5* | *exon17:c.C3047T* | *p.Thr1016Met* | *base substitution* | *VUS* | *0.00004153* |
| *NO.32* | *TTN* | *NM_001267550.2* | *exon239:c.44174_44176del* | *p.14725_14726del* | *deletion* | *VUS* | *--* |
|  | *TTN* | *NM_001267550.2* | *exon49:c.G14339A* | *p.Ser4780Asn* | *base substitution* | *VUS* | *--* |
|  | *PRKAG2* | *NM_016203.4* | *exon3:c.C425T* | *p.Thr142Ile* | *base substitution* | *VUS* | *0.000060* |
| *NO.33* | *TTN* | *NM_001267550.2* | *exon339:c.C93254T* | *p.Pro31085Leu* | *base substitution* | *VUS* | *0.0000698* |
|  | *RBM20* | *NM_001134363.3* | *exon9:c.G1999T* | *p.Ala667Ser* | *base substitution* | *VUS* | *0.000001289* |
|  | *FLNC* | *NM_001458* | *exon20:c.G3100A* | *p.Gly1034Arg* | *base substitution* | *VUS* | *--* |
| *NO.34* | *TTN* | *NM_001267550.2* | *exon9:c.C1412T* | *p.Ala471Val* | *base substitution* | *VUS* | *--* |
|  | *ACTN2* | *NM_001103.4* | *exon8:c.C710T* | *p.Thr237Ile* | *base substitution* | *VUS* | *0.000001303* |
|  | *JUP* | *NM_002230.4* | *exon9:c.C1550T* | *p.Pro517Leu* | *base substitution* | *VUS* | *0.00002* |
| *NO.35* | *TTN* | *NM_001267550.2* | *exon358:c.A101251C* | *p.Asn33751His* | *base substitution* | *VUS* | *--* |
|  | *RYR2* | *NM_001035.3* | *exon38:c.T5774C* | *p.Ile1925Thr* | *base substitution* | *VUS* | *0.00003532* |
|  | *RBM20* | *NM_001134363.3* | *exon13:c.G3545A* | *p.Arg1182His* | *base substitution* | *VUS* | *0.00009281* |
| *NO.36* | *TTN* | *NM_133379* | *exon32 c.7468C>T* | *p.Arg2490Cys* | *base substitution* | *VUS* | *--* |
|  | *KIF20A* | *NM_005733.3* | *exon2 c.100G>A* | *p.Val34Met* | *base substitution* | *VUS* | *--* |
| *NO.37* | *TTN* | *NM_001267550.2* | *exon91:c.G26464A* | *p.Ala8822Thr* | *base substitution* | *VUS* | *0.0001* |
| *NO.38* | *TTN* | *NM_001267550.2* | *exon326:c.G82810A* | *p.Gly27604Ser* | *base substitution* | *VUS* | *--* |
|  | *ACTN2* | *NM_001103.4* | *exon18:c.C2240T* | *p.Ala747Val* | *base substitution* | *VUS* | *0.000002478* |
|  | *MYH7* | *NM_000257.4* | *exon7:c.T602C* | *p.Ile201Thr* | *base substitution* | *VUS* | *--* |
|  | *XIRP2* | *NM_152381.6* | *exon9:c.G3001A* | *p.Val1001Ile* | *base substitution* | *VUS* | *0.0005943* |
| *NO.39* | *TTN* | *NM_001267550.2* | *exon326: c.78466G>A* | *p.Asp26156Asn* | *base substitution* | *VUS* | *--* |
|  | *TTN* | *NM_001267550.2* | *exon221: c.40634-19A>C* |  | *base substitution* | *VUS* | *--* |
|  | *TTN* | *NM_001267550.2* | *exon49:c.14339G>A* | *p.Ser4780Asn* | *base substitution* | *VUS* | *0.0011* |
|  | *PKP2* | *NM_004572.4* | *exon2: c.224-3C>G* |  | *base substitution* | *VUS* | *--* |
|  | *LAMP2* | *NM_002294.3* | *exon3: c.380C>T* | *p.Pro127Leu* | *base substitution* | *VUS* | *0.0000095746* |
| *NO.40* | *TTN* | *NM_001267550.2* | *exon157: c.35366C>A* | *p.Pro11789His* | *base substitution* | *VUS* | *0.00009722* |
|  | *TTN* | *NM_001267550.2* | *exon155: c.34931-7C>T* |  | *base substitution* | *VUS* | *0.00007146* |
|  | *BAG3* | *NM_004281.4* | *exon3: c.605C>T* | *p.Pro202Leu* | *base substitution* | *VUS* | *0.0000837614* |
|  | *BAG3* | *NM_004281.4* | *exon3: c.626C>T* | *p.Pro209Leu* | *base substitution* | *LP* | *--* |
| *NO.41* | *TTN* | *NM_133378.4* | *exon229: c.46444C>T* | *p.Arg15482Cys* | *base substitution* | *VUS* | *0.0079* |
|  | *LAMA4* | *NM_002290.5* | *exon2: c.122C>T* | *p.Ala41Val* | *base substitution* | *VUS* | *0.0011* |
|  | *RBM20* | *NM_001134363.3* | *exon10: c.2565_2570del* | *p.Gln856_Glu857del* | *deletion* | *VUS* | *0.0025* |
| *NO.42* | *TTN* | *NM_133378.4* | *exon195: c.37856G>A* | *p.Gly12619Asp* | *base substitution* | *VUS* | *0.00005642* |
|  | *RYR2* | *NM_001035.3* | *exon37: c.5096G>A* | *p.Arg1699His* | *base substitution* | *VUS* | *0.0000209333* |
| *NO.43* | *TTN* | *NM_133378.4* | *exon265: c.58964T>C* | *p.Met19655Thr* | *base substitution* | *VUS* | *0.000008066* |
|  | *TTN* | *NM_133378.4* | *exon151: c.31378G>T* | *p.Val10460Phe* | *base substitution* | *VUS* | *0.0002* |
|  | *DYSF* | *NM_003494.4* | *exon31: c.3352G>A* | *p.Gly1118Ser* | *base substitution* | *VUS* | *0.00002784* |
|  | *DYSF* | *NM_003494.4* | *exon43: c.4742G>A* | *p.Arg1581His* | *base substitution* | *VUS* | *0.0034* |
| *NO.44* | *TTN* | *NM_133378.4* | *exon126: c.28711A>G* | *p.Lys9571Glu* | *base substitution* | *VUS* | *0.00002791* |
|  | *MYH7* | *NM_000257.4* | *exon23: c.2723T>C* | *p.Leu908Pro* | *base substitution* | *VUS* | *--* |
| *NO.45* | *TTN* | *NM_133378.4* | *exon275: c.78167G>A* | *p.Arg26056His* | *base substitution* | *VUS* | *0.0001* |
|  | *TTN* | *NM_133378.4* | *exon215: c.42536A>G* | *p.Asp14179Gly* | *base substitution* | *VUS* | *0.000004044* |
|  | *RBM20* | *NM_001134363.3* | *exon13: c.3545G>A* | *p.Arg1182His* | *base substitution* | *VUS* | *0.0025* |
| *NO.46* | *TTN* | *NM_001267550.2* | *exon3: c.244G>A* | *p.Ala82Thr* | *base substitution* | *VUS* | *--* |
|  | *FLNC* | *NM_001458.5* | *exon19: c.2879T>G* | *p.Val960Gly* | *base substitution* | *VUS* | *0.0002* |
| *NO.47* | *TTN* | *NM_001267550.2* | *exon348: c.97070C>T* | *p.Thr32357Ile* | *base substitution* | *VUS* | *0.0002* |
|  | *TNNI3* | *NM_000363.5* | *exon8: c.575G>A* | *p.Arg192His* | *base substitution* | *LP* | *--* |
|  | *MYLK2* | *NM_033118.4* | *exon9: c.1292G>A* | *p.Arg431Gln* | *base substitution* | *VUS* | *0.0003* |
|  | *FLNC* | *NM_001458.4* | *exon25: c.4301G>A* | *p.Arg1434His* | *base substitution* | *VUS* | *0.000174425* |
| *NO.48* | *TTN* | *NM_001267550.2* | *exon358: c.101395A>G* | *p.Thr33799Ala* | *base substitution* | *VUS* | *0.0002* |
|  | *ALPK3* | *NM_020778.4* | *exon5: c.1385A>G* | *p.His462Arg* | *base substitution* | *VUS* | *0.000004062* |
|  | *RBM20* | *NM_001134363.3* | *exon2: c.593C>T* | *p.Pro198Leu* | *base substitution* | *VUS* | *--* |
| *NO.49* | *TTN* | *NM_001267550.2* | *exon49: c.14146C>G* | *p.Leu4716Val* | *base substitution* | *VUS* | *--* |
|  | *TTN* | *NM_001267550.2* | *exon48: c.11669A>G* | *p.Tyr3890Cys* | *base substitution* | *VUS* | *--* |
|  | *PKP2* | *NM_004572.4* | *exon2: c.256T>C* | *p.Tyr86His* | *base substitution* | *VUS* | *--* |
| *NO.50* | *TTN* | *NM_001267550.2* | *exon315: c.66388G>C* | *p.Val22130Leu* | *base substitution* | *VUS* | *0.0001* |
|  | *KCNH2* | *NM_000238.4* | *exon10: c.2483G>A* | *p.Cys828Tyr* | *base substitution* | *VUS* | *--* |
| *NO.51* | *TTN* | *NM_001267550.2* | *exon257: c.48284G>A* | *p.Arg16095Gln* | *base substitution* | *VUS* | *0.000031904* |
|  | *TTN* | *NM_001267550.2* | *exon98: c.28257C>T* | *p.His9419=* | *base substitution* | *LB* | *0.0000120574* |
|  | *MYH7* | *NM_000257.4* | *exon16: c.1603G>C* | *p.Glu535Gln* | *base substitution* | *LP* | *--* |
|  | *PRDM16* | *NM_022114.4* | *exon9: c.1840G>A* | *p.Asp614Asn* | *base substitution* | *VUS* | *0.000111495* |
| *NO.52* | *TTN* | *NM_001267550.2* | *exon326: c.74510G>A* | *p.Gly24837Asp* | *base substitution* | *VUS* | *0.00005623* |
|  | *SCN5A* | *NM_000335.5* | *exon28: c.4856C>A* | *p.Thr1619Lys* | *Base substitution* | *VUS* | *--* |
| *NO.53* | *TTN* | *NM_001267550* | *exon335:c.A90164T* | *p.Asp30055Val* | *base substitution* | *VUS* | *--* |
|  | *PSEN2* | *NM_000447.3* | *exon6:c.T437C* | *p.Ile146Thr* | *base substitution* | *VUS* | *0.000004339* |
|  | *SDHA* | *NM_004168.4* | *exon8:c.G986A* | *p.Arg329Gln* | *base substitution* | *VUS* | *0.00001115* |

P,pathogenic;LP,likely pathogenic;VUS,variant of uncertain significance;LB,likely benign.
